# DNA-like class R inhibitory oligonucleotides (INH-ODNs) preferentially block autoantigen-induced B-cell and dendritic cell activation *in vitro *and autoantibody production in lupus-prone MRL-Fas^*lpr*/*lpr *^mice *in vivo*

**DOI:** 10.1186/ar2710

**Published:** 2009-05-28

**Authors:** Petar Lenert, Kei Yasuda, Liliana Busconi, Patrice Nelson, Courtney Fleenor, Radhika S Ratnabalasuriar, Peter L Nagy, Robert F Ashman, Ian R Rifkin, Ann Marshak-Rothstein

**Affiliations:** 1 Departments of Internal Medicine and Pathology, Carver College of Medicine, The University of Iowa, Iowa City, C312GH, 200 Hawkins Drive, IA 52242, USA; 2 Departments of Microbiology and Medicine, Boston University School of Medicine, Boston, 715 Albany Street, MA 02118, USA

## Abstract

**Introduction:**

B cells have many different roles in systemic lupus erythematosus (SLE), ranging from autoantigen recognition and processing to effector functions (for example, autoantibody and cytokine secretion). Recent studies have shown that intracellular nucleic acid-sensing receptors, Toll-like receptor (TLR) 7 and TLR9, play an important role in the pathogenesis of SLE. Dual engagement of rheumatoid factor-specific AM14 B cells through the B-cell receptor (BCR) and TLR7/9 results in marked proliferation of autoimmune B cells. Thus, strategies to preferentially block innate activation through TLRs in autoimmune B cells may be preferred over non-selective B-cell depletion.

**Methods:**

We have developed a new generation of DNA-like compounds named class R inhibitory oligonucleotides (INH-ODNs). We tested their effectiveness in autoimmune B cells and interferon-alpha-producing dendritic cells *in vitro *and in lupus-prone MRL-Fas^*lpr*/*lpr *^mice *in vivo*.

**Results:**

Class R INH-ODNs have 10- to 30-fold higher inhibitory potency when autoreactive B cells are synergistically activated through the BCR and associated TLR7 or 9 than when stimulation occurs via non-BCR-engaged TLR7/9. Inhibition of TLR9 requires the presence of both CCT and GGG triplets in an INH-ODN, whereas the inhibition of the TLR7 pathway appears to be sequence-independent but dependent on the phosphorothioate backbone. This difference was also observed in the MRL-Fas^*lpr*/*lpr *^mice *in vivo*, where the prototypic class R INH-ODN was more effective in curtailing abnormal autoantibody secretion and prolonging survival.

**Conclusions:**

The increased potency of class R INH-ODNs for autoreactive B cells and dendritic cells may be beneficial for lupus patients by providing pathway-specific inhibition yet allowing them to generate protective immune response when needed.

## Introduction

Nucleic acids, including self DNA and RNA, are recognized by a subset of Toll-like receptors (TLRs) [1-4]. To discriminate between self and non-self nucleic acids, the nucleic acid-sensing TLRs 3, 7, 8, and 9 are expressed only within the cell interior, contrasting with other TLRs (for example, TLR2 or TLR4) that are expressed on cell surfaces. Upon ligand entry into the cell, TLR9 migrates from the endoplasmic reticulum to CpG-DNA-containing endosomes [5,6]. Interestingly, the type of endosomal compartment to which TLR9 relocates depends on cell type and the nature of the TLR ligand used for activation. For example, in the response of human dendritic cells (DCs) to linear CpG-DNA, TLR9 activation goes through late LAMP-1-positive endosomes [7,8]. In contrast, stimulation with complex TLR9 ligands is more restricted in terms of responding cell types and, in DCs, proceeds through early endosomes instead. The uptake of these complex ligands may be facilitated by CXCL16, which may influence this differential compartmentalization [9]. Interestingly, the outcome of the DC response to TLR9 stimulation varies greatly depending on where TLR9 meets CpG-DNA. For example, type I interferon-alpha (IFN-α) secretion is induced by complex class A(D) CpG-oligonucleotides (CpG-ODNs) via early endosomal signaling, whereas interleukin-6/tumor necrosis factor-alpha (IL-6/TNF-α) secretion requires late endosomal signaling and is induced primarily by linear TLR9 ligands [8].

Although bacterial DNA and double-stranded CpG-ODNs stimulate macrophages vigorously, they are very poor activators of resting B cells in both humans and mice [10-13]. In resting follicular B cells and in human naïve peripheral blood B cells, engagement of the B-cell receptor (BCR) for antigen, together with co-stimulation with either type I/II IFN or BAFF (B-cell activating factor of TNF family), may prime B cells to overcome this unresponsiveness to complex TLR ligands [13-18]. This enhancement may be due to multiple mechanisms (for example, TLR7 and 9 upregulation, increased ligand uptake, BCR-mediated delivery of TLR ligands to 'autophagosomes' where concomitant BCR and TLR signals take place, or lowered BCR signaling threshold) [19]. It remains to be formally proven whether the same type of the crosstalk between BCR and TLR also occurs between antigen and co-delivered TLR7 ligand.

These findings have direct implications for the pathogenesis of systemic lupus erythematosus (SLE), a multisystemic disease in which autoantibodies to DNA- and RNA-containing autoantigens (for example, nucleosomes, Ku-autoantigen, Sm/RNP, or splicesosomes) are the immunologic hallmark of the disease [20-22]. These antibodies frequently antedate the clinical disease, and high levels of several lupus autoantibodies nicely correlate with either specific disease subsets (for example, lupus nephritis, congenital heart block, or subacute cutaneous lupus) or disease activity in general [20,23]. Immune complexes between complement-fixing anti-double-stranded DNA (anti-dsDNA) antibodies and corresponding autoantigens are held responsible for the kidney damage in lupus nephritis [20]. Complement levels frequently fall during major lupus flares, further suggesting that complement-activating immune complexes may play an important role in the tissue pathology [20].

It was recently found that lupus autoantigens (for example, nucleosomes or Sm/RNP) have intrinsic 'autoadjuvant' activities (endogenous mitogens) when complexed with corresponding autoantibodies, causing proliferation of autoreactive B cells and type I IFN secretion from plasmacytoid DCs [24-32]. Depending on the nature of the initiating autoantigen, the proliferation requires either the TLR7 or TLR9 pathway, including the presence of the key adaptor protein MyD88 (myeloid differentiation primary response gene 88) [25-28,33,34]. Thus, therapies aimed at blocking the TLRs may be beneficial for treating lupus. Indeed, promising results have been reported in animal models of lupus using TLR7- and/or TLR9-specific antagonists [35-37].

We have recently developed a new class of inhibitory ODNs that we named class R ('restricted') INH-ODNs [38]. We show that these dsDNA-like analogues carrying the canonical TLR9-inhibitory sequence [39,40] are selective for certain autoreactive B cells and for type I IFN-producing DCs. They are 10- to 30-fold less potent in non-autoreactive B cells stimulated with linear CpG-DNA ligands. In addition to autoreactive B cells, class R INH-ODNs are capable of blocking both DNA/anti-DNA-induced and RNA/anti-RNA-induced IFN-α secretion from DCs. Interestingly, the latter outcome is ODN sequence-independent but is dependent on a nuclease-resistant phosphorothioate (PS) backbone. Class R INH-ODNs are also active *in vivo*, where they preferentially block anti-dsDNA and anti-Sm/RNP antibody secretion and prolong survival of lupus-prone mice. Refinement of the class R INH-ODN structure to combine optimal TLR7/TLR9 sequences in double-stranded carrier may result in a novel class of pathway-specific therapeutics for human lupus.

## Materials and methods

### Creating class R inhibitory ODNs for the Toll-like receptor-9 signaling pathway

We used INH-ODN 4084-F, with a PS backbone, as a template for creating class R INH-ODNs (Table 1). INH-ODN 4084-F is the shortest active 12-mer INH-ODN that contains both previously identified suppressive elements (CCT and GGG), appropriately spaced from each other (four nucleotides apart) and properly oriented in a single-stranded ODN molecule (5'-CCT → GGG-3') [39]. INH-1 was created by adding a complementary strand of nucleotides generating a 24-mer ODN forming a complete palindrome. INH-18 is a linear 24-mer class B INH-ODN in which the 5' INH-ODN 4084-F sequence was followed by a random stretch of 12 nucleotides lacking the ability to form significant secondary structures. ODNs INH-43, INH-45, and INH-47 are palindromic variants of INH-1 in which the CCT and/or GGG elements were replaced with random nucleotide triplets. Similarly, ODNs INH-44, INH-46, and INH-48 are linear derivatives of INH-18 lacking CCT, GGG, or both triplets. INH-4 and INH-13 are palindromic or linear analogues of INH-1 and INH-18, with the difference that the canonical CCT and GGG blocks are placed at the 3' end of the molecule. We have further created INH-ODNs, based on either INH-1 (for 5') or INH-4 (for 3') as templates, in which the complementary/non-CCT/GGG-containing half was truncated to create hairpin structures with short (three nucleotides), medium (six nucleotides), or long (nine nucleotides) 5' or 3' overhangs. Shortened linear derivatives of INH-18 and INH-13 were synthesized to serve as controls for palindromic INH-ODNs with 3' and 5' overhangs. Importantly, neither the complementary sequence to 4084-F nor the random nucleotide sequence in the 3' half of INH-18 showed any inhibitory activity on TLR9-stimulated B cells or macrophages at concentrations as high as 1 μM (data not shown).

**Table 1:** Synthetic Toll-like receptor-9 agonists and antagonists used in the study

	TLR9-antagonists	Class	Properties
4084-F	CCT GGA TGG GAA	B	5' linear, shortest active
INH-1	CCT GGA TGG GAA TTC CCA TCC AGG	R	5' palindrome
INH-18	CCT GGA TGG GAA CTT ACC GCT GCA	B	5' linear
INH-43	CCT GGA TAA AAA TTT TTA TCC AGG	R	Lacks GGG
INH-44	CCT GAA TAA AAA CTT ACC GCT GCA	B	Lacks GGG
INH-45	TAT GGA TGG GAA TTC CCA TCC ATA	R	Lacks CCT
INH-46	TAT GGA TGG GAA CTT ACC GCT GCA	B	Lacks CCT
INH-47	TAT GGA TTT TAA TTA AAA TCC ATA	R	Lacks CCT/GGG
INH-48	TAT GGA TTT TAA CTT ACC GCG GCA	B	Lacks CCT/GGG
5' OVHG-short	CCT GGA TGG GAA TTC CCA TCC	R	5' short overhang (3 nucleotide OVHG)
5' OVHG-scr. short	CCT GGA TGG GAA CTT ACC GCT	B	5' linear
5' OVHG-medium	CCT GGA TGG GAA TTC CCA	R	5' medium overhang (6 nucleotide OVHG)
5' OVHG-scr. medium	CCT GGA TGG GAA CTT ACC	B	5' linear
5' OVHG-long	CCT GGA TGG GAA TTC	R	5' long overhang (9 nucleotide OVHG)
5' OVHG-scr. long	CCT GGA TGG GAA CTT	B	5' linear
INH-4 3' palindrome	TTC CCA TCC AGG CCT GGA TGG GAA	R	3' palindrome
INH-13 3' scr. pal.	CTT ACC GCT GCA CCT GGA TGG GAA	B	3' linear
3' OVHG-short	CCA TCC AGG CCT GGA TGG GAA	R	3' short overhang (3 nucleotide OVHG)
3' OVHG-scr. short	ACC GCT GCA CCT GGA TGG GAA	B	3' linear
3' OVHG-medium	TCC AGG CCT GGA TGG GAA	R	3' medium overhang (6 nucleotide OVHG)
3' OVHG-scr. medium	GCT GCA CCT GGA TGG GAA	B	3' linear
3' OVHG-long	AGG CCT GGA TGG GAA	R	3' long overhang (9 nucleotide OVHG)
3' OVHG-scr. long	GCA CCT GGA TGG GAA	B	3' linear
4173	RRR RRR RRR RRR RRR	B	Linear control, R = random
	TLR9-agonists	Class	Properties
CpG-2336	ggGGACGACGTCGTGgggggg	A(D)	Lowercase PS linkages
CpG-1826	TCC ATG ACG TTC CTG ACG TT	B(K)	Linear, murine
CpG-2006	TCG TCG TTT TGT CGT TTT GTC GTT	B(K)	Linear, human

OVHG, overhang; pal., palindrome; PS, phosphorothioate; scr., scrambled; TLR, Toll-like receptor.

### Toll-like receptor agonists

CpG-ODNs 2336, 1826, and 2006 (Table 1) were obtained from Coley Pharmaceuticals (Ottawa, ON, Canada). All other ODNs were synthesized and HPLC (high-performance liquid chromatography)-purified by Integrated DNA Technologies (IDT) (Coralville, IA, USA) and used at concentrations of up to 1 μM (for PS-ODNs) or 30 μM (for phosphodiester [PO]-ODNs). PS-ODNs have a PS backbone, and PO-ODNs have a native PO backbone. TLR7/8 ligands R-837, CL-075, CL-097, and loxoribine were purchased from InvivoGen (San Diego, CA, USA). All reagents were endotoxin-free as determined by the Limulus amebocyte lysate assay (Pyrotell LAL Assay; Associates of Cape Cod, Inc., East Falmouth, MA, USA). Highly purified lipopolysaccharide (LPS) was obtained from List Biological Laboratories, Inc. (Campbell, CA, USA).

### Animal studies

MRL-MpJ-Fas^*lpr*/*lpr *^mice were purchased from The Jackson Laboratory (Bar Harbor, ME, USA) and maintained under standard conditions in the Animal Facility at The University of Iowa. AM14 mice, expressing a BCR reactive with mouse IgG2a, were described previously [41-43] and were bred and maintained in microisolator cages at the Laboratory Animal Science Center of the Boston University School of Medicine. Several cohorts of young pre-diseased MRL-Fas ^*lpr*/*lpr *^mice were treated beginning from 4 weeks (rederived strain, MRL-MpJ-Fas^lpr^/J) or 15 weeks of age (non-rederived strain, MRL-MpJ-Fas^lpr^/2J). Phosphate-buffered saline (PBS), INH-1, INH-18, INH-47, or INH-48 (1 mg/kg body weight) in a final volume of 1 mL was injected intraperitoneally or subcutaneously three times weekly for 12 to 25 weeks. Each experimental group consisted of five to eight female mice. At the beginning and at the end of the treatment protocol, blood was obtained through retro-orbital bleeds and urine was collected. Serum was analyzed for cytokines, total immunoglobulin (Ig) levels, and autoantibodies. Proteinuria was semi-quantified using Multistix urinalysis strips (Bayer, Leverkusen, Germany). The study was approved by the University of Iowa animal ethics committee, and animals were euthanized according to Institutional Animal Care and Use Committee procedures. Left kidney, liver, and lungs/heart blocks were harvested and fixed in 10% buffered formalin. Paraffin-embedded organ sections were stained with periodic acid-Schiff and hematoxylin/eosin. The extent of kidney damage was graded according to published guidelines and scored in a blinded fashion [35]. The right kidney was embedded in Sakura Finetek Tissue-Tek O.C.T. compound (Sakura Finetek U.S.A., Inc., Torrance, CA, USA) and kept frozen at -80°C before use in immunohistology for detection of C3 and IgG deposits.

### INH-ODN potency studies in primary macrophages, macrophage cell lines, and human and mouse B cells

Splenic macrophages were obtained from C57BL6 mice by recovering the CD43^+ ^fraction from the magnetic-activated cell sorting (MACS) LD columns. Cells were left to adhere to plastic for 4 hours. The adherent fraction typically contained greater than 85% CD11b^+^F4/80^+ ^cells. Experiments were repeated with similar results using splenic macrophages obtained by positive selection using CD11b microbeads and two rounds of positive selection (>97% purity). For B-cell enrichment, the pass-through CD43^- ^MACS fraction was used as a source of total B cells. The purity of B-cell fraction was typically greater than 97% as judged by CD19/B220 fluorescence-activated cell sorting staining. The ratio between the CD21^int^CD23^+ ^follicular B cells and CD21^high^CD23^low/- ^marginal zone B cells was approximately 8:1 to 15:1 in control strains and in young (4-week-old) MRL-^*lpr*/*lpr *^mice. However, with age, this ratio became substantially lower in the lupus strain [13].

For INH-ODN potency studies, enriched primary macrophages, total splenic B cells, RAW264 macrophages, and the human B-cell line (Namalwa) were incubated for 18 to 42 hours with optimal concentrations of class A(D) (100 nM), class B(K) stimulatory CpG-ODNs (10 to 33 nM), TLR7/8 ligands R-837 (1 μg/mL), or CL-075 (0.1 μg/mL) plus increasing concentrations of INH-ODNs (from 1 to 1,000 nM). B-cell cycle entry and protection from spontaneous apoptosis was detected using acridine orange flow cytometry as described previously [44]. Cell culture supernatants were collected and tested for cytokines in enzyme-linked immunosorbent assay (ELISA). For determination of polyclonal IgM, B cells were cultured for 6 days.

### DNA – or RNA- immune complex-stimulated AM14 B cells and dendritic cells

B cells were isolated from AM14 BCR transgenic mice by positive selection using anti-B220-coupled magnetic beads [17]. AM14 B cells were stimulated with the IgG2a monoclonal antibodies (mAbs) PL2-3, as a form of chromatin-containing immune complexes [25], or BWR4 (10 μg/mL) (kindly provided by Dan Eilat, Hadassah University Hospital, Jerusalem, Israel) as a form of RNA immune complexes [28]. Results were confirmed by stimulating AM14 B with anti-Sm antibody Y2 (20 μg/mL final concentration) (kindly provided by Philip Cohen and Robert Eisenberg, University of Pennsylvania, Philadelphia, PA, USA) pre-mixed with purified endotoxin-free Sm/RNP (0.628 mg/mL). For the BWR4 and Y2 studies, B cells were pre-treated with IFN-α (PBL Laboratories) (1,000 U/mL IFN-α for 2 to 3 hours at 37°C) to upregulate TLR7 expression, resulting in markedly enhanced proliferation upon stimulation with BWR4 antibodies. In some experiments, AM14 B cells were also stimulated with linear CpG B(K) ODN-1826 (0.3 μg/mL) or ultrapure LPS (List Biological Laboratories, Inc.).

Control ODN 4173, INH-1, INH-18, and their variants were added to cultures simultaneously with the DNA or RNA immune complexes. Cell proliferation was measured after 24 hours by pulsing B cells for an additional 6 hours with [^3^H] thymidine. On their own, Sm/RNP particles had no activity in B cells. Non-transgenic B cells, in contrast to AM14 B cells, failed to proliferate to BWR4 antibodies, whereas TLR9-deficient AM14 B cells still proliferated, thus ruling out cross-reactivity with DNA in culture fluids.

Bone marrow-derived DCs from Balb/c mice were cultured with the Flt-3L for 8 days. Highly enriched DCs (3 × 10^4 ^cells per 200 μL volumes) were additionally cultured over the course of a 24-hour period with DNA immune complexes containing CG50 (dsDNA fragment derived from the plasmid pMCG-50 containing 50 repeats of CpG and used at 100 ng/mL [26]) combined with PA4 IgG2a anti-dsDNA antibodies (kindly provided by Mark Monestier, Temple University, Philadelphia, PA, USA) (10 μg/mL). INH-ODNs were pre-incubated with DCs for 30 minutes before adding immune complexes. In parallel experiments, class A(D) CpG-ODN 2336 was used for stimulation instead (at a concentration of 300 nM). Supernatants were collected and IFN-α measured in ELISA.

For TLR7-dependent stimulation of DCs, RNA immune complexes were made by using the 05-02A antibody preparation (SLE1) [45] from a human lupus patient at 50 μg/mL. Control ODN, INH-1 and INH-18, and their variants lacking the CCT and/or GGG were added to RNA/anti-RNA-stimulated DC cultures at a concentration of 1 μg/mL. IFN-α was measured after 24 hours.

### Enzyme-linked immunosorbent assay and immunofluorescence studies for autoantibody detection

Serum samples from PBS- or INH-ODN-treated mice were diluted appropriately and tested for antinuclear and anti-dsDNA antibodies using HEp-2 or *Crithidia luciliae*-coated slides, respectively (Inova Diagnostics, Inc., San Diego, CA, USA). Bound IgG was revealed using fluorescein isothiocyanate-labeled anti-mouse IgG (Bethyl Laboratories, Inc., Montgomery, TX, USA). Specific staining of kinetoplasts on Crithidia slides, detected by immunofluorescence on an Olympus BX-51 microscope (Olympus, Tokyo, Japan), was used as a criterion for the presence of anti-dsDNA antibodies in lupus sera.

Autoantibody levels against dsDNA and Sm/RNP were further quantified by ELISA. Calf thymus Sm/nRNP antigen was purchased from ImmunoVision (Springdale, AR, USA). Ultrapure calf-thymus dsDNA was from Sigma-Aldrich (St. Louis, MO, USA). For anti-dsDNA detection, polystyrene plates were pre-coated with poly-L-lysine followed by S_1 _nuclease-treated dsDNA (coated at 5 μg/mL). Lupus sera were diluted 1:500 and incubated on dsDNA-coated plates for 30 minutes at room temperature. After extensive washings, bound IgG was detected using horseradish peroxidase (HRP)-labeled anti-mouse IgG1, IgG2a, or IgM antibodies (Bethyl Laboratories, Inc.), respectively. A similar procedure was used for the detection of anti-Sm/RNP antibodies on plates coated with 2.5 μg/mL Sm/nRNP.

### Cytokine enzyme-linked immunosorbent assay

IL-6, TNF-α, and IL-12p40 were detected using pairs of antibodies obtained from eBioscience, Inc. (San Diego, CA, USA). For detection of type I IFN secretion [45], we used rat anti-mouse IFN-α mAb (22100-1; PBL Biomedical Laboratories, Piscataway, NJ, USA), rabbit anti-mouse IFN-α polyclonal antibody (32100-1; PBL Biomedical Laboratories), HRP-conjugated donkey anti-rabbit IgG (711-036-152; Jackson ImmunoResearch Laboratories, Inc., West Grove, PA, USA), and mouse r-IFN-α as a standard (12100-1; PBL Biomedical Laboratories). The detection limit of the IFN-α ELISA was 80 pg/mL.

## Results

### Prototypic class R and class B INH-ODNs have similar potencies and efficacies in macrophages and bone marrow-derived dendritic cells

**Figure 1:**
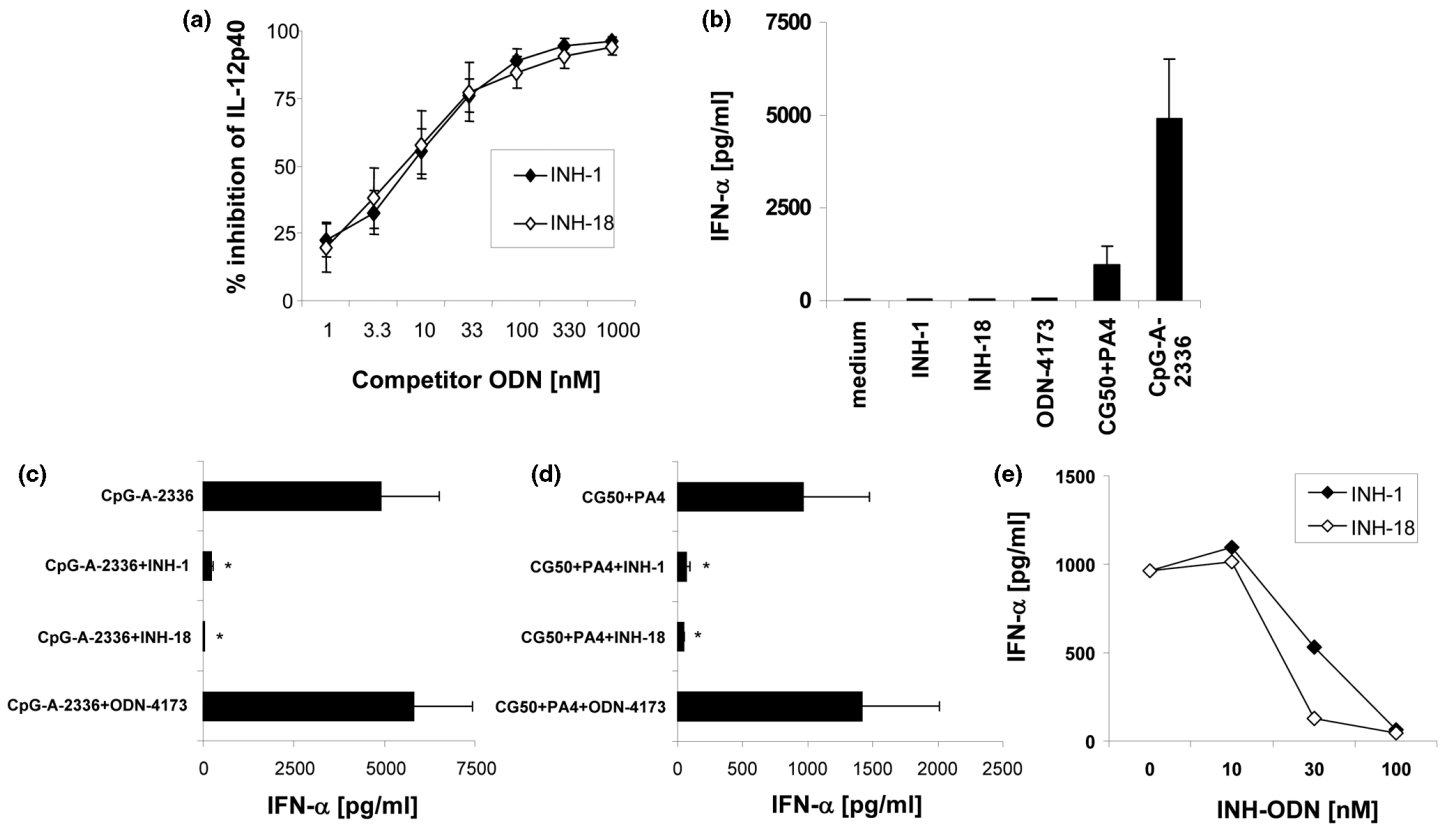
Class R and B inhibitory oligonucleotides (INH-ODNs) have similar inhibitory potencies for Toll-like receptor-9 (TLR9)-activated primary macrophages and dendritic cells (DCs). **(a) **Enriched primary macrophages were stimulated with class A(D) CpG-ODN (100 nM) for 18 to 24 hours. INH-ODNs were added over the concentration range shown. Interleukin (IL)-12p40 and tumor necrosis factor-alpha (TNF-α) were measured in enzyme-linked immunosorbent assay (ELISA). Flt-3-propagated bone marrow-derived DCs were stimulated for 24 hours either with class A(D) CpG-ODN 2336 or CG50+PA4 immune complexes or with various combinations of TLR9 ligands and class R or class B INH-ODNs or control ODNs. INH-ODNs and control ODNs were used either at a concentration of 1 μg/mL **(b-d) **or over the concentration range shown **(e)**. Interferon-alpha (IFN-α) secretion was measured in ELISA (n = 3 to 5). *P < 0.05.

Enriched primary macrophages from C57BL6 mice (1 × 10^6 ^per well) (Figure 1a) were stimulated with a class A(D) CpG-ODN, with INH-ODNs added simultaneously. INH-1 (class R INH-ODN, palindromic) and INH-18 (class B INH-ODN, linear) were used over the concentration range shown. Cell culture supernatants were collected after 18 hours and analyzed for IL-12p40 (macrophages) by ELISA. Inhibition by INH-1 and INH-18 was identical.

Bone marrow-derived Flt-3L-propagated DCs secreted IFN-α in response to the class A(D) stimulatory CpG-ODN 2336 and gave a smaller response to CG50/PA4 immune complexes. Neither class of INH-ODNs nor control ODNs induced measurable IFN-α secretion (Figure 1b). When added to DC cultures simultaneously with TLR9 ligands, class R (INH-1) and class B (INH-18) INH-ODNs (but not the control ODN) showed similar inhibitory potency for TLR9 ligand-induced IFN-α production (Figure 1c–e). Figure 1e shows the dose response for inhibition of CG50+PA4-induced IFN-α secretion.

### Palindromic INH-ODNs with phosphorothioate backbones show 10- to 30-fold lower potency than linear INH-ODNs for inhibiting Toll-like receptor-9 stimulation of primary mouse and human B cells

**Figure 2:**
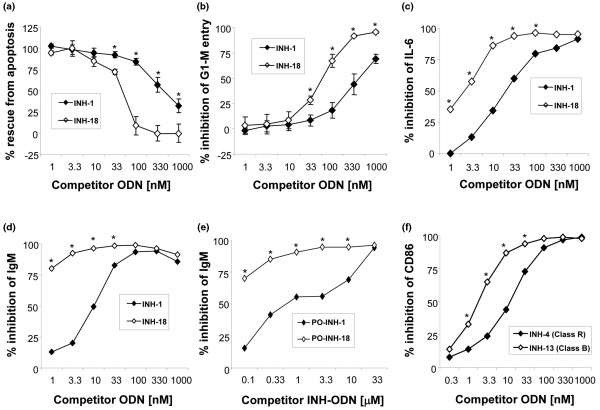
Class R inhibitory oligonucleotides (INH-ODNs) show lower potency for resting mouse and human B cells. Total CD43^- ^B cells from C57BL6 mice were stimulated with class B(K) CpG-ODN 1826 for either 18 to 42 hours **(a-c) **or 6 days **(d) **in the presence of increasing concentrations of INH-ODNs (1 to 1,000 nM). Percentage of cells with hypodiploid DNA content and cells entering the G_1_-M phase of the cell cycle was determined in acridine orange flow cytometry. Interleukin-6 (IL-6) and polyclonal IgM were measured in enzyme-linked immunosorbent assay (ELISA) (n = 3 to 7). **(e) **Total mouse B cells were stimulated with PO-CpG-ODN for 6 days. **(f) **Total human Namalwa B cells were stimulated with human PS-CpG-2006 for 42 hours. CD86 expression and polyclonal IgM secretion were measured. Indicated class R and B INH-ODNs were added over the concentration range shown (n = 3 or 4). *P < 0.05. PO, phosphodiester; PS, phosphorothioate.

We next tested the effect of these INH-ODNs on resting mouse B cells stimulated with linear TLR9 ligands. Total CD43^- ^resting B cells from 6- to 8-week-old C57BL6 spleens (composed of approximately 90% follicular B cells and approximately 10% marginal zone B cells) were stimulated with linear CpG-1826 either for 18 to 24 hours (for measuring cell cycle entry, apoptosis protection, and IL-6 secretion) or for 6 days (for polyclonal IgM secretion). A range of concentrations of either class B or class R INH-ODNs were added simultaneously. While both INH-ODNs showed efficacy, linear class B INH-18 was 10- to 30-fold more potent than class R INH-1, even though different outcome assays required different concentrations of INH-ODNs to reach the 50% inhibition point (for example, Apo>G1-M entry>IL-6>IgM) (Figure 2a–d).

To check whether the observed difference extends to INH-ODNs synthesized with the natural PO backbone, INH-1 and INH-18 were made with the PO backbone. In this case, we used a stimulatory CpG-ODN also made with the PO backbone at a concentration of 10 μM. Various outcomes (for example, IL-6, G_1_-M entry, and IgM secretion) were measured, and dose-dependent inhibition of IgM secretion is shown. Again, at least a 30-fold potency difference for IgM secretion between PO backbone versions of INH-1 and INH-18 was observed (Figure 2e).

Similar to resting mouse B cells, the human B-cell line Namalwa, expressing a high level of TLR9 [46], was sensitive to inhibition with both class R and class B INH-ODNs (Figure 2f) when CD86 upregulation was measured as an outcome. However, again class B INH-ODNs (INH-13 is shown) were 10 times more potent than class R (INH-4) (Figure 2f). Similar results were observed in human peripheral blood B cells and with INH-1 compared with INH-18 (data not shown).

### The size of the single-stranded overhang in INH-ODNs with partial palindromes determines the potency difference between class B and class R INH-ODNs

**Figure 3:**
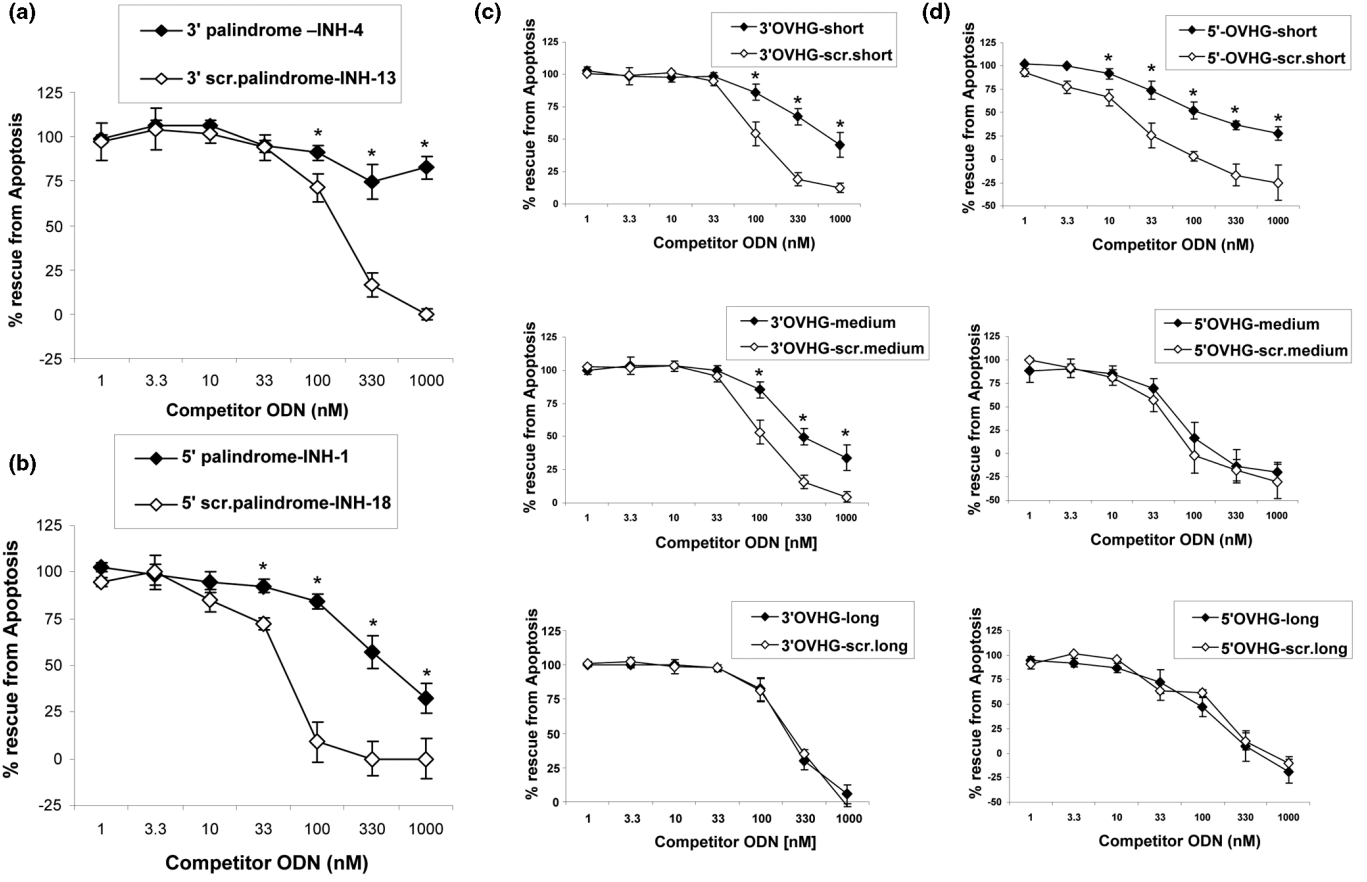
The size of the linear overhang determines the potency difference between class R and class B inhibitory oligonucleotides (INH-ODNs). Total mouse B cells were stimulated with 33 nM CpG-1826 together with indicated class R or class B INH-ODNs added simultaneously and used over the concentration range shown. **(a) **Palindromic Class R INH-4 and linear INH-13 ODNs with CCT/GGG blocks at the 3' were used. **(b) **Palindromic Class R INH-1 and linear INH-18 ODNs with CCT/GGG blocks at the 5' end were used. **(c) **INH-ODNs with short, medium or long 3' linear overhangs or linear INH-ODNs of the equal length were used. **(d) **INH-ODNs with short, medium or long 5' linear overhangs or linear INH-ODNs of the equal length were used. Inhibition of CpG-1826-induced rescue from apoptosis is shown. Sequences of INH-ODNs are shown in Table 1 (n = 3 to 5). *P < 0.05. OVHG, overhang; scr., scrambled.

We next created INH-ODNs with partial palindromes and single-stranded linear segments at their 3' or 5' ends ranging from three to nine nucleotides in length. We reasoned that the selectivity favoring linear INH-ODNs in resting B cells may be lost if 5'→3' or 3'→ 5' helicases are recruited to the TLR9 signalosome. As predicted, creating INH-ODNs with progressively longer linear overhangs attached to their 3' or 5' ends increased the potency of such class R INH-ODNs in TLR9-stimulated B cells, eventually abrogating the difference between the class B and class R INH-ODNs. Figure 3 shows results of apoptosis protection; however, very similar data were observed with other B-cell outcomes (for example, G_1_-M entry, IL-6, and IgM secretion).

### Class R INH-ODNs require canonical CCT and GGG triplets for inhibition of rheumatoid factor-specific AM14 B cells stimulated with DNA-containing immune complexes

**Figure 4:**
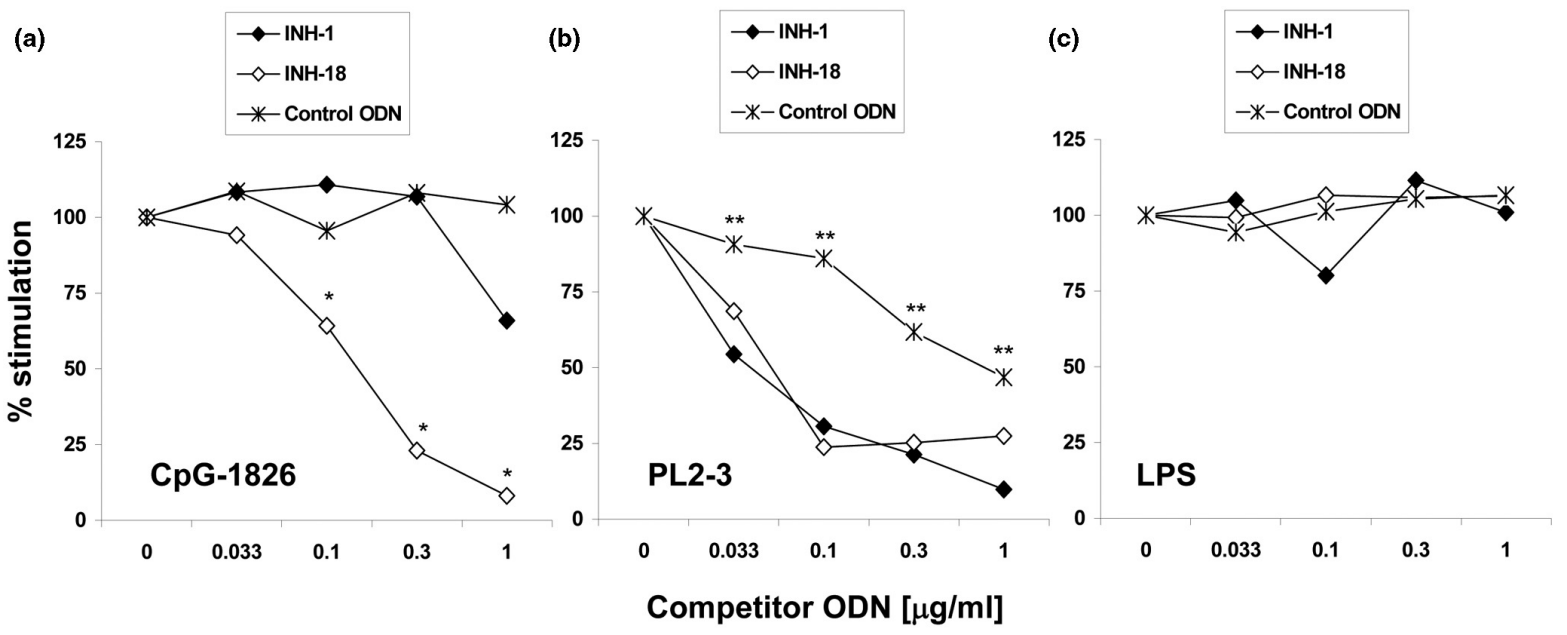
Higher potency of class R inhibitory oligonucleotides (INH-ODNs) for B-cell receptor-dependent activation of AM14 B cells induced with DNA-containing immune complexes. AM14 B cells were stimulated with **(a) **linear CpG-1826, **(b) **anti-nucleosome antibody PL2-3, or **(c) **lipopolysaccharide (LPS), and class R and class B INH-ODNs were added simultaneously. Proliferation of AM14 B cells was determined by measuring the [^3^H] thymidine incorporation for the last 6 hours. Results are expressed as percentage of maximal stimulation induced with the particular Toll-like receptor ligand (n = 3). *P < 0.05 (INH-1 versus INH-18). **P < 0.05 (INH-1 versus control).

A very useful model for studying autoreactive B-cell activation, the AM14 B cells express transgenic BCRs that recognize IgG2a antibodies. When PL2-3 (IgG2a) anti-nucleosome antibodies are added to spent cultures, AM14 B cells proliferate in a TLR9-dependent fashion [25]. Similar to resting mouse B cells, AM14 cells proliferate in response to linear TLR9 ligands (CpG-1826) and this proliferation can be inhibited in a dose-dependent fashion with class B INH-18 but not with control ODNs (Figure 4a). Class R INH-1 is at least 10-fold less potent than INH-18 when CpG-1826 is used for stimulation. However, when PL2-3 antibodies are used for stimulation, the potency of class R INH-ODNs increases 10-fold to equal that of class B INH-ODNs (Figure 4b). Since the control ODNs showed slight inhibitory activity, it means that added ODNs compete with endogenous DNA either for binding to PL2-3 antibodies or for the subsequent BCR-mediated delivery to a TLR9 signaling compartment. As a control for specificity and to rule out non-selective toxicity of INH-ODNs, we also stimulated AM14 B cells with LPS. Neither INH-ODNs nor control ODNs could inhibit LPS-induced AM14 proliferation over the concentration range shown in Figure 4c.

**Figure 5:**
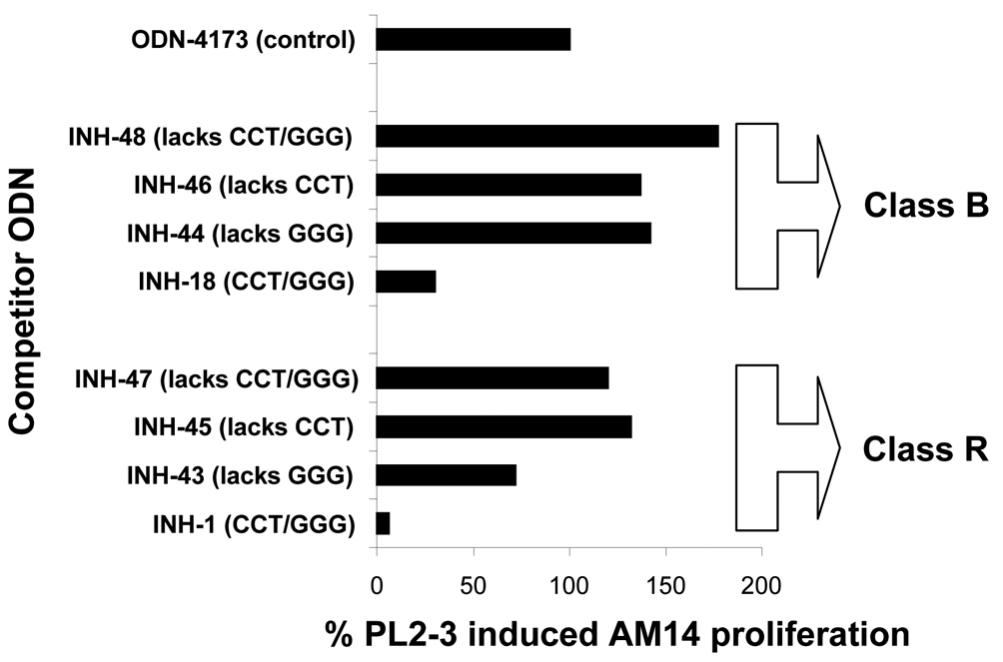
Both classes of inhibitory oligonucleotides (INH-ODNs) require CCT and GGG triplets for the full inhibitory activity. AM14 B cells were stimulated with PL2-3 immune complexes. Indicated INH-ODNs and control ODN-4173 (all at a concentration of 1 μg/mL) were added simultaneously. Proliferation was measured. One of two similar experiments is shown.

We next tested sequence requirements for inhibition in AM14 B cells. We created linear (18, 44, 46, and 48) and palindromic (1, 43, 45, and 47) INH-ODNs lacking the CCT element (45 and 46), GGG element (43 and 44), or both (47 and 48). Similar to control ODN 4173, INH-47 and INH-48 not only lacked the ability to block the TLR9 stimulation of AM14 B cells induced with PL2-3-containing immune complexes but actually co-stimulated proliferation to a certain degree (Figure 5). The two elements were equally important for inhibition, as observed both in class B and in class R INH-ODN variants tested, suggesting that the requirement for both CCT and GGG elements previously observed in non-autoreactive B cells also applies to AM14 B cells.

### INH-ODNs made with phosphorothioate backbone inhibit activation of dendritic cells, macrophages, and RNA/anti-RNA-stimulated AM14 B cells in a sequence-independent and backbone-dependent fashion

**Figure 6:**
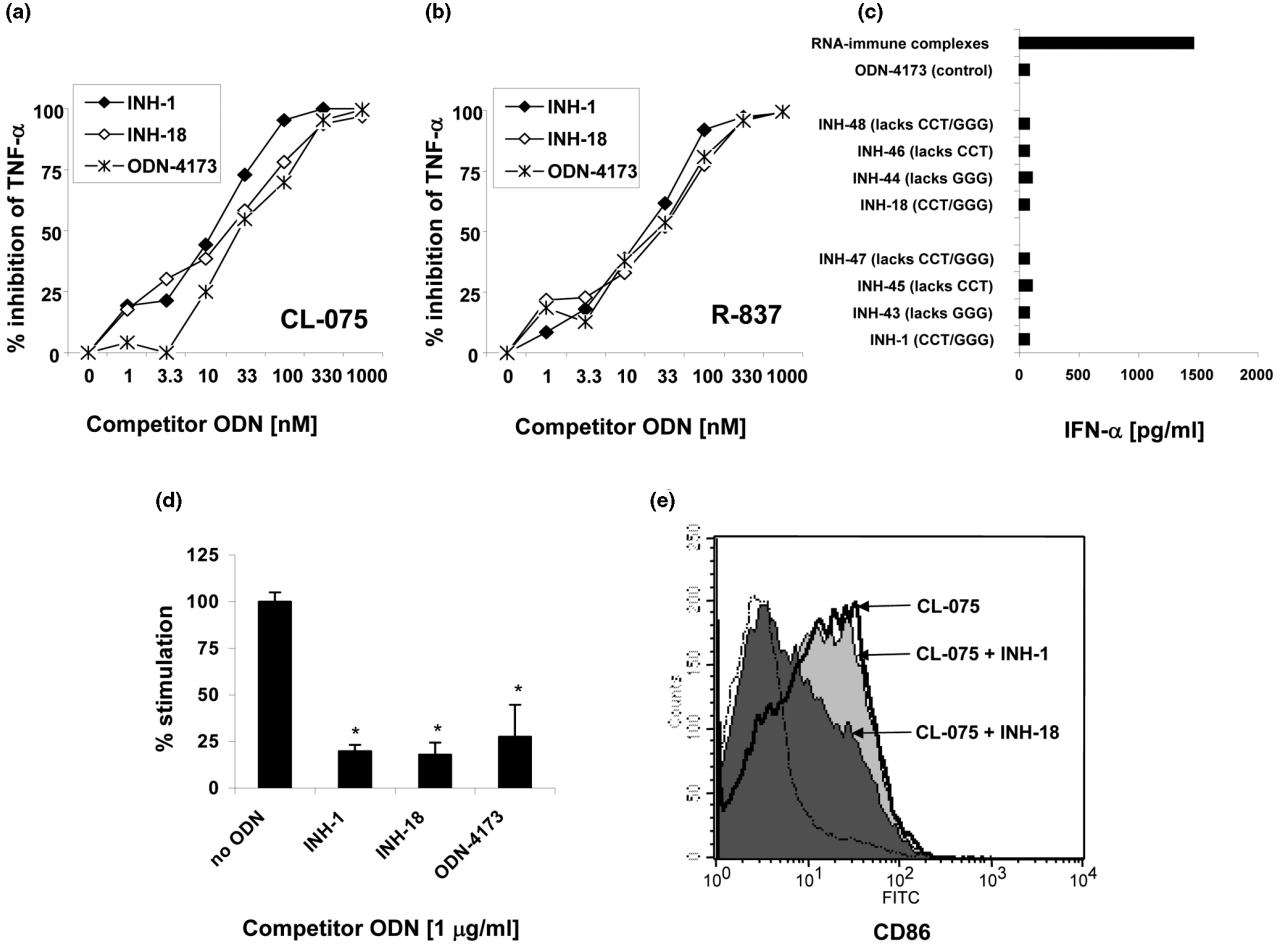
Class R and B inhibitory oligonucleotides (INH-ODNs) inhibit Toll-like receptor-7 (TLR7)-dependent activation of macrophages, dendritic cells (DCs), AM14 B cells, and primary mouse B cells in a sequence-independent but backbone-dependent manner. RAW264.7 macrophages **(a, b)**, Flt-3L-propagated bone marrow-derived DCs **(c)**, AM14 B cells **(d)**, and primary mouse resting B cells **(e) **were stimulated with TLR7/8 ligands (CL-075, R-837, or RNA immune complexes as indicated) with INH-ODNs or control ODNs added simultaneously. Tumor necrosis factor-alpha (TNF-α) and interferon-alpha (IFN-α) were measured in enzyme-linked immunosorbent assay. AM14 proliferation was determined by measuring the [^3^H] thymidine incorporation for the last 6 hours. CD86 expression was determined by flow cytometry (n = 3 to 5). *P < 0.05 (ODN-treated versus medium-treated samples). FITC, fluorescein isothiocyanate.

Recent literature suggests that some TLRs may bind their ligands through their sugar backbone residues and that possession of a PS backbone not only protects from nucleases but also increases affinity for the relevant TLR [3,4]. As TLR7 has restricted cell distribution in humans (plasmacytoid DCs and B cells) and has been linked to lupus pathogenesis in mice [47-49], we next tested the ability of class R and B INH-ODNs to block TLR7-induced activation of macrophages, DCs, AM14 B cells, and primary mouse B cells. Figures 6a and 6b show the ability of both classes of INH-ODNs to block the activation of RAW macrophages in a dose-dependent fashion. Fifty percent inhibition was achieved with approximately 10 to 33 nM. These results were obtained with TLR7 ligands R-837 and CL-075, respectively. Interestingly, the same level of inhibition was observed with the control ODN-4173. Similar results were observed in primary mouse macrophages (data not shown). We next studied the effect of class R and B INH-ODNs and their variants on TLR7 activation of bone marrow-derived Flt-3L-propagated DCs. IFN-α secretion was measured in ELISA. When ODNs were used at a concentration of 1 μg/mL (approximately 125 nM), RNA/anti-RNA-induced IFN-α secretion was similarly inhibited, not only by prototypic class B and class R INH-ODNs, but also by their variants lacking CCT and/or GGG, as well as control ODN-4173 (Figure 6c). We further studied the effect of INH-ODNs 1 and 18 and the control ODN 4173 on BWR4 (IgG2a) + RNA-induced proliferation of AM14 B cells (Figure 6d). Similarly to macrophages and DCs, stimulation of AM14 B cells with RNA-containing immune complexes (including immune complexes containing the lupus autoantigen Sm/RNP and anti-Sm antibodies; data not shown) was inhibited indiscriminately with both classes of INH-ODNs and with control ODNs. However, in primary non-autoreactive mouse B cells, INH-18, compared with INH-1, was a much more potent inhibitor of the TLR7 ligand-induced CD86 expression (Figure 6e). When INH-ODNs were made with natural PO backbone and tested at concentrations of up to 10 μM, neither class B nor class R INH-ODNs could inhibit R-837-induced activation of RAW cells or IgM secretion from primary B cells (data not shown), suggesting a PS backbone-dependent inhibitory effect on TLR7 activation, not affected by base sequence.

### Class R INH-ODNs block anti-double-stranded DNA and anti-Sm/RNP secretion *in vivo *in lupus mice

**Figure 7:**
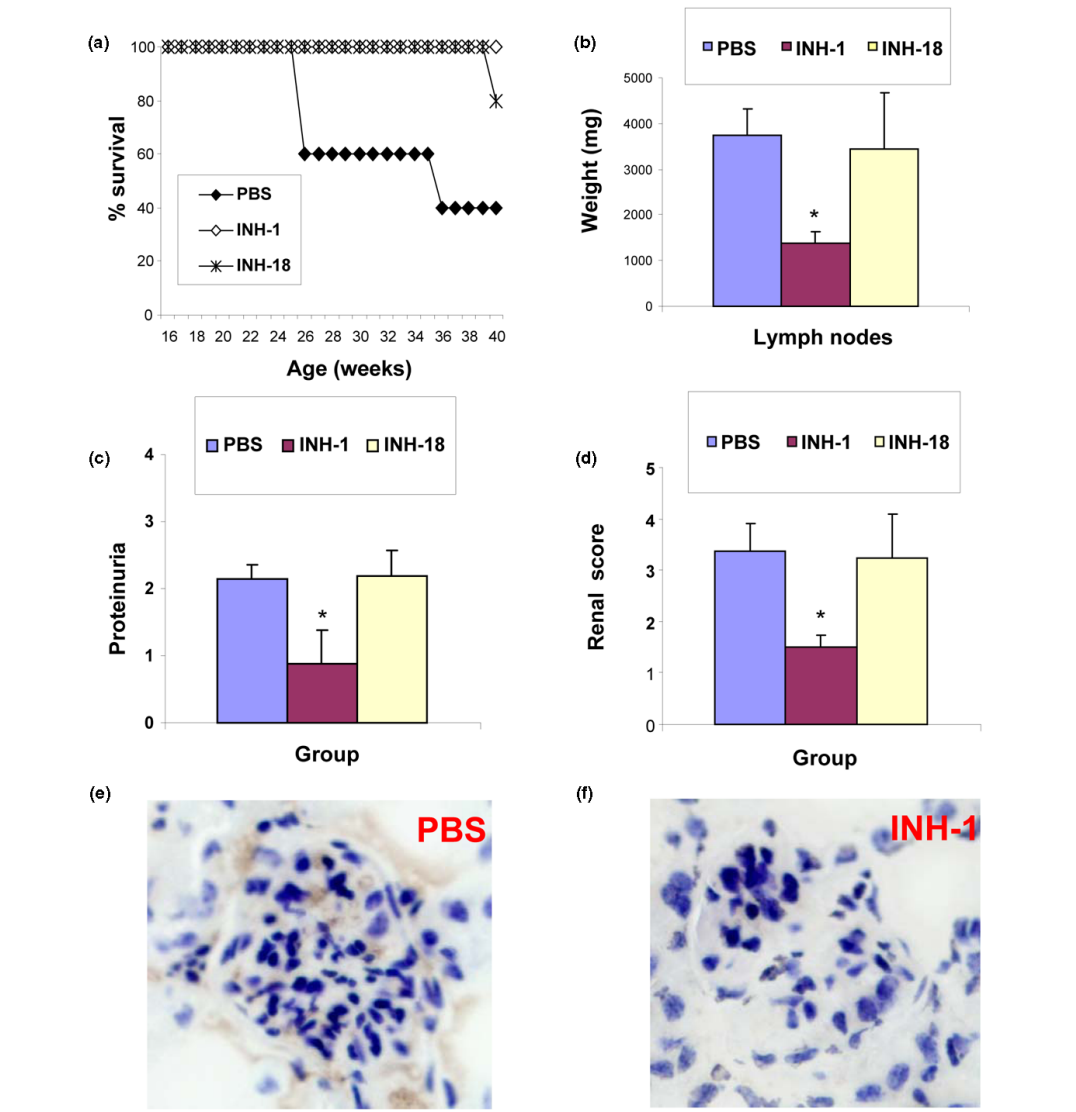
Inhibitory oligonucleotides (INH-ODNs) prolong survival and decrease morbidity in MRL-Fas^*lpr*/*lpr *^mice *in vivo*. Pre-diseased MRL-Fas^*lpr*/*lpr *^(2J strain) mice were treated with INH-1, INH-18, or vehicle starting from week 15 for 25 consecutive weeks. ODNs were injected intraperitoneally three times weekly at the concentration of 1 mg/kg body weight. Surviving mice were sacrificed at week 40. Similar results were obtained in three additional cohorts of lupus mice (J and 2J strains). Effects of INH-ODNs on survival **(a)**, total lymph node weight **(b)**, proteinuria **(c)**, composite renal score **(d)**, and IgG deposits in kidneys **(e, f) **are shown. *P < 0.05 (ODN-treated versus vehicle-treated). PBS, phosphate-buffered saline.

**Figure 8:**
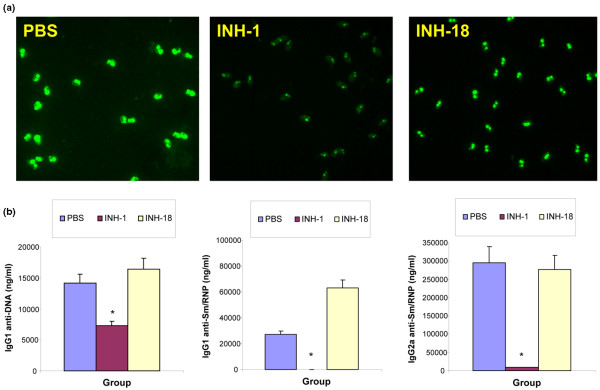
Class R inhibitory oligonucleotides (INH-ODNs) preferentially block autoantibody secretion in MRL-Fas^*lpr*/*lpr *^mice *in vivo*. Pre-diseased MRL-Fas^*lpr*/*lpr *^mice were treated with INH-1, INH-18, or vehicle for 25 weeks as in Figure 7. Surviving mice were sacrificed and their sera tested for autoantibodies. **(a) **The presence of anti-double-stranded DNA (anti-dsDNA) antibodies binding native DNA was determined semi-quantitatively by staining the *Crithidia lucillae *kinetoplasts. **(b) **The concentrations of anti-DNA and anti-Sm/RNP antibodies were further measured in enzyme-linked immunosorbent assay. *P < 0.05 (ODN-treated versus vehicle-treated). PBS, phosphate-buffered saline.

To address whether class R INH-ODNs preferentially affect autoreactive B cells *in vivo *more than normal B cells, we used the MRL-Fas^*lpr*/*lpr *^model of lupus. In this model, mice develop massive lymphoproliferation (lymphadenomegaly and splenomegaly) due to the mutation in the Fas and additionally produce numerous autoantibodies, including anti-dsDNA and anti-Sm/RNP [50]. Several lines of evidence suggest that autoreactive B cells play a primary role in the pathogenesis of SLE, not only as autoantibody secreting effector cells but as key antigen-presenting cells [23]. When pre-diseased MRL-Fas^*lpr*/*lpr *^mice (2J strain) were treated with either class R or class B INH-ODNs intraperitoneally at a dose of 1 mg/kg three times weekly for 25 weeks, INH-ODN-treated lupus mice survived longer than vehicle-treated controls (Figure 7a). INH-1-treated, but not INH-18-treated, mice also showed less lymphadenomegaly (Figure 7b), less proteinuria (Figure 7c), and decreased composite renal score (Figure 7d). As shown in Figures 7e and 7f, INH-1-treated mice displayed less prominent glomerular and peritubular IgG deposits. Furthermore, palindromic class R INH-ODNs were more effective than linear class B INH-ODNs as inhibitors of both anti-dsDNA antibody secretion (detected by immunofluorescence and by ELISA) and of anti-Sm/RNP antibody secretion (as detected by ELISA) (Figure 8).

## Discussion

It was first observed by Pisetsky and his group [51,52] that several G-rich ODNs made with the PS, but not PO, backbone had inhibitory activity. We and others [39,40,53-61] have extended these early observations to define structural requirements for TLR9 stimulation and inhibition in the nanomolar range. Strikingly, changes at the 5' end of an ODN (either inhibitory or stimulatory), particularly those affecting the pyrimidine-rich CCT triplet, diminished both the stimulatory and inhibitory activity for the TLR9 pathway [39,62]. We have also identified a stretch of three (four) consecutive Gs necessary for optimal inhibition in the nanomolar range [39,40,63].

Could DNA-like therapeutics offer a pathway-specific tool for treating systemic lupus? Variants of our prototypic INH-ODN 2114 (TCCTGGAGGGGAAGT) [44,64] have already been tested in lupus-prone animals. For example, Patole and colleagues [36] found that INH-ODN 2114 was active in the MRL-^*lpr*/*lpr *^strain, whereas Klinman's group [35] discovered that an ODN containing multiple TTAGGG repeats, like those in telomeric DNA, was inhibitory in lupus-prone NZB/NZW F1 mice. Barrat and colleagues [37] recently obtained similar results in the NZB/W-F1 strain, with an INH-ODN that combined the canonical TLR9-inhibitory motif with a TLR7-specific TGC triplet at the 5' end. One dsDNA-based biocompound LJP 394 (Abetimus) developed by the La Jolla Pharmaceutical Company (San Diego, CA, USA) has already entered clinical trials in human lupus and showed some promising results (for example, ability to decrease anti-dsDNA antibody production *in vivo*) [65].

The current report addresses the relevance of secondary structure to INH-ODN activity. We created class R INH-ODNs by starting with the shortest strongly active 12-mer linear INH-ODN 4084-F (CCTGGATGGGAA) (Table 1) and then extending it with 12 more bases, resulting in complete palindromes (class R) or in non-palindromic linear sequences (class B). Class R INH-ODNs were between 10- to 30-fold less potent as inhibitors in resting mouse B cells than class B INH-ODNs when synthetic linear TLR9 agonists were used for stimulation (Figure 2). Similar findings were obtained in human primary B cells/B-cell lines. In contrast to resting B cells, when 'professional antigen-presenting cells' (for example, primary macrophages and bone marrow-derived DCs) were stimulated either with complex CpG-class A(D) ODNs or with DNA-containing immune complexes, classes R and B INH-ODNs showed very similar inhibitory potencies (Figure 1). Stimulation with these complex TLR9 ligands is believed to occur in DCs in an early endosomal compartment [7,8].

This difference in inhibitory activity between class B and class R INH-ODNs in resting (follicular) B cells was clearly dependent on the ability of INH-ODNs to make double-stranded secondary structures. The biggest decrease in potency was observed with class R INH-ODNs containing either complete palindromes or short (up to three nucleotides) single-stranded 5' or 3' linear overhangs (Figure 3). Further increase in the length of the single-stranded overhang progressively reduced the difference between linear and class R INH-ODNs.

These results suggest a hypothesis that, in B cells (though not in macrophages), dsDNA (including class R INH-ODNs) has limited access to endosomal TLR9 relative to single-stranded DNA. Single-stranded CpG-ODNs have been shown to encounter TLR9 in LAMP-1-positive endosomes [8,9]. We postulated that, if dsDNA gains access to these endosomes by BCR-mediated entry, the difference in potencies between double-stranded and single-stranded versions of the same inhibitory sequence would disappear. This hypothesis could be tested in transgenic B cells expressing a BCR either for dsDNA or for Ig by stimulating the cells with one or both of these components.

Thus, important proofs of concept emerged from studies in AM14 B cells (Figures 7 and 8). These rheumatoid factor-specific B cells express a transgenic BCR derived from hybridoma-secreting anti-Ig-specific autoantibodies [43]. Their BCR recognizes the Fc-fragment of IgG2a antibodies expressing 'a' but not 'b' allotype [25]. *In vitro*, AM14 B cells proliferate when anti-nucleosome antibodies (PL2-3) are added to spent cultures (Figure 4). This proliferation is sensitive to DNAse treatment and requires an intact MyD88/TLR9 pathway [25,34], clearly arguing for a role of endogenous DNA in PL2-3-induced AM14 B-cell activation. Similar to other TLR9-expressing cells, AM14 B cells proliferate vigorously to stimulation with linear CpG ligands (Figure 4a).

These results suggest that PL2-3 binds nucleosome-associated chromatin released into the cultures from dying cells and that these immune complexes engage the anti-Ig BCR, deliver 'signal 1', and enter the cell [25]. The dsDNA they contain then reaches TLR9 in the endosome ('autophagosomes' [18]), otherwise accessible only to single-stranded DNA. Therefore, AM14 B cells represent an excellent model that can be used to contrast data obtained through BCR-dependent and BCR-independent TLR9-mediated signaling and to test some predictions of this hypothesis. As in non-autoreactive B cells, our results show that 'BCR-independent' stimulation with linear CpG-ODN 1826 was differentially inhibited by class R and class B INH-ODNs, with class B 10-fold more potent than class R (Figure 4a). However, in 'BCR-dependent' immune complex-induced B-cell proliferation, classes B and R INH-ODNs showed similar potencies for inhibition, primarily because of the increased potency of class R INH-ODNs for BCR-mediated activation (Figure 4b).

While the exact mechanism of class R INH-ODNs action in autoreactive B cells remains to be determined, several possibilities may be considered: (a) competition between TLR9-inhibiting ODNs and endogenous DNA for binding to PL2-3 antibodies/nucleosome/HMGB1 complex, (b) competition for binding to membrane RAGE (receptor for advanced glycation endproducts)/BCR complex, (c) preferential recruitment of TLR9 into early versus late endosomes depending on the nature of the TLR9 ligand used, (d) BCR-dependent increase in passive endocytosis of class R INH-ODNs, and (e) BCR-dependent recruitment of specific helicases (DNA-unzipping enzymes) into an early endosomal compartment. These possibilities are not mutually exclusive. For example, inhibition of BCR signaling might result from both better uptake/trafficking of class R INH-ODNs into endosomes and better recruitment of helicases into TLR9-containing compartments. Helicases must have an important role in the class R INH-ODN-mediated inhibition because recent affinity studies revealed superior binding of single-stranded TLR9 ligands to chip-immobilized TLR9 [3], an event that may depend heavily on the sugar backbone of the TLR9 ligands used for stimulation [4].

Another possibility is a direct competition between endogenous DNA and INH-ODNs for binding to anti-dsDNA antibodies. While this possibility may account for some competition observed in the PL2-3 model, there are no data in the literature to suggest that natural anti-dsDNA antibodies (or anti-chromatin antibodies) preferentially bind DNA segments carrying the canonical inhibitory motif over other DNA sequences [33].

The possibility that the double-stranded structure of class R INH-ODNs may direct these ODNs preferentially to an early endosomal compartment in macrophages and DCs and into BCR-related 'autophagosomes' in autoreactive B cells has a precedent in a work by Guiducci and colleagues [8]. These authors have found that IFN-α secretion from human plasmacytoid DCs induced by multimeric class A(D) CpG-ODNs occurs in transferring receptor-positive endosomes whereas linear monomeric CpG-ODNs (like CpG-1826 used in our experiments) preferentially localize to LAMP-1-positive endosomes and are poor stimulators of IFN-α secretion. However, when linear CpG ligands are complexed into microparticles, they now gain the ability to move to transferrin receptor-positive endosomes, inducing robust IFN-α production. Therefore, the most important determinant of TLR9 signaling may be its endosomal localization [7,8] or alternatively CXCL16 engagement as suggested by the Klinman's group [9]. For one or more of these reasons, class R INH-ODNs have higher potency for BCR-dependent TLR9 activation in autoreactive B cells than for BCR-independent activation of normal B cells.

In this article, we further question the ability of 'TLR9-specific' INH-ODNs to target additional TLR signaling pathways. While our previous data [44] and data from several other groups [60,61] have clearly identified that INH-ODNs at concentrations of up to 1 μM fail to inhibit signaling through the TLR2, 3, and 4 pathways, recent literature has suggested a possibility that PS-ODNs, including INH-ODNs, may also block the TLR7 signaling pathway [28]. It is believed that, in mice (in contrast to humans), the TLR8 pathway is non-functional since cells from TLR7^-/- ^mice fail to respond to stimulation with synthetic TLR7/8 ligands [2]. Here, we show that our INH-ODNs can block TLR7-dependent activation of primary macrophages, macrophage cell lines, RNA immune complex-activated DCs, and AM14 B cells in a dose-dependent but sequence-independent manner (Figure 6a–d). However, in spite of these sequence-independent inhibitory effects, palindromic INH-ODNs still have lower potency for resting B cells (Figure 6e), but classes B and R are equally potent and effective in RNA/anti-RNA-stimulated AM14 B cells (Figure 6d). Here again, BCR engagement is associated with greater potency of class R INH-ODNs.

Finally, *in vivo*, in the MRL-Fas^*lpr*/*lpr *^strain, class R INH-ODNs were better than class B INH-ODNs as inhibitors of both IgG anti-dsDNA and anti-Sm/RNP antibody secretion (Figure 8). These results may have significant impact for developing novel DNA-like therapeutics for treating lupus. While TLR7^-/- ^MRL-Fas^*lpr*/*lpr *^lupus mice have better survival, they still succumb to autoimmune disease [49], thus suggesting a possible role for additional DNA/RNA-triggered intracellular signaling pathways (for example, DAI and RIG-I). Interestingly, in this strain, complete lack of TLR9 results in two opposite outcomes: diminished anti-dsDNA secretion but increased anti-Sm/RNP antibody production [49]. Moreover, the survival of these mice is reduced, likely due to insufficient number/function of regulatory T cells [66]. One can wonder whether the ability of class R INH-ODNs to block both TLR9 and TLR7 activation in autoreactive B cells may explain the beneficial effect of these INH-ODNs on autoantibody secretion *in vivo*. The inferior efficacy of more *in vitro *potent class B INH-ODNs in lupus mice came as a surprise. While we lack a logical explanation for this result, recent literature suggests that PS-ODNs (including TLR9-specific INH-ODNs) may redirect TLR7/8 ligand-induced activation away from the TLR7 toward the TLR8 pathway, as observed in HEK cells expressing TLR7 and TLR8 and in primary human peripheral blood mononuclear cells [67] and mouse cells [68]. This redirection of TLR activation may result in decreased IFN-α secretion from plasmacytoid DCs but a corresponding increase in IL-12, TNF-α, and IFN-γ secretion resulting from TLR8-expressing cells (for example, human monocytes) [67].

Future studies should explore the efficacy of class R INH-ODNs in different strains of lupus mice, including the BXSB male mice. This lupus strain harbors a duplication of the TLR7 gene which appears to be responsible for the phenotype [47,48]. Moreover, refinement of the palindromic structure to generate combined selective TLR7/TLR9 inhibitors [37] together with anti-B-cell-depleting protocols [23] to re-establish critical B-cell differentiation checkpoints [69] may result in better treatments for human lupus.

## Conclusions

Class R INH-ODNs have a high potency for autoreactive B cells and DCs *in vitro *and are effective in the MRL-Fas^*lpr*/*lpr *^model of lupus *in vivo*.

## Abbreviations

BCR: B-cell receptor; DC: dendritic cell; dsDNA: double-stranded DNA; ELISA: enzyme-linked immunosorbent assay; HRP: horseradish peroxidase; IFN: interferon; Ig: immunoglobulin; IL: interleukin; INH-ODN: inhibitory oligonucleotide; LPS: lipopolysaccharide; mAb: monoclonal antibody; MACS: magnetic-activated cell sorting; MyD88: myeloid differentiation primary response gene 88; ODN: oligonucleotide; PBS: phosphate-buffered saline; PO: phosphodiester; PS: phosphorothioate; SLE: systemic lupus erythematosus; TLR: Toll-like receptor; TNF: tumor necrosis factor.

## Competing interests

The authors declare that they have no competing interests.

## Authors' contributions

PL conceived the study, participated in the design, and helped to draft the manuscript and has given final approval of the version to be published. KY carried out studies in DCs. LB and PN carried out studies in AM14 B cells. CF and RSR carried out studies in MRL mice. PLN was involved in animal data analysis. RFA helped with the study design and coordination in B cells and drafted the manuscript. IRR participated in the design of DC studies. AM-R participated in AM14 B-cell studies and drafted the manuscript. All authors read and approved the final manuscript.
